# Use-inspired basic research on individual differences in face identification: implications for criminal investigation and security

**DOI:** 10.1186/s41235-018-0115-6

**Published:** 2018-06-27

**Authors:** Karen Lander, Vicki Bruce, Markus Bindemann

**Affiliations:** 1 Division of Neuroscience and Experimental Psychology, School of Biological Sciences, University of Manchester, Manchester, M13 9PL UK; 2 School of Psychology, Newcastle University, Newcastle, UK; 3 School of Psychology, University of Kent, Canterbury, UK

**Keywords:** Face identification, Face recognition, Face matching, Individual differences, Passport control, Super-recognisers

## Abstract

This journal is dedicated to “use-inspired basic research” where a problem in the world shapes the hypotheses for study in the laboratory. This review considers the role of individual variation in face identification and the challenges and opportunities this presents in security and criminal investigations.

We show how theoretical work conducted on individual variation in face identification has, in part, been stimulated by situations presented in the real world. In turn, we review the contribution of theoretical work on individual variation in face processing and how this may help shape the practical identification of faces in applied situations. We consider two cases in detail. The first case is that of security officers; gatekeepers who use facial ID to grant entry or deny access. One applied example, where much research has been conducted, is passport control officers who are asked to match a person in front of them to a photograph shown on their ID. What happens if they are poor at making such face matching decisions and can they be trained to improve their performance? Second, we outline the case of “super-recognisers”, people who are excellent at face recognition. Here it is interesting to consider whether these individuals can be strategically allocated to security and criminal roles, to maximise the identification of suspects.

We conclude that individual differences are one of the largest documented sources of error in face matching and face recognition but more work is needed to account for these differences within theoretical models of face processing.

## Significance

There is a huge volume of laboratory-based and more applied work on face matching and recognition, where the focus has been on average performance measures from groups of participants. This work has generally ignored the variance in performance in individuals. We discuss how applied situations on face matching and recognition have added to the growing impetus for theoretical work on individual variation in face identification. In particular we consider passport control officers and their ability to make face matching decisions. We also highlight super-recognisers with their excellent ability to make accurate recognition decisions. Theoretical work on individual variation may impact the practical procedures, recruitment and policies in criminal and security roles that require face identification skills.

## Background

Most research on face processing has used a group approach whereby performance on a face task is averaged across participants, ignoring the differences in performance between individuals. Extensive group-mean research has explored the circumstances under which people may generally do well, or not so well, at identifying faces in laboratory or simulated eyewitness experiments (for example, Johnston & Edmonds, 2009). However, it is also known that there is huge variation in individual face identification ability.^1^ For example, in the Glasgow Face Matching Test (Burton, White, & McNeill, 2010), where participants are simply asked to decide if two photographs of faces belong to the same person or different people, performance varied from almost chance (53%) to perfect (100%). A similar pattern of huge variation is inherent across many face perception and recognition tasks and within different participant populations. Indeed, whilst most people have a relatively similar amount of experience with faces, there is a considerable range in their face identification performance. At the lower end of performance some individuals are significantly impaired at face identification. Until relatively recently, it was rare cases of “prosopagnosia”, acquired as a result of brain injury or disease, that were investigated (see Grusser & Landis, 1991 for a review). In the past 20 years, however, there has been considerable interest in a much larger group of people, who appear always to have had problems identifying faces, termed “developmental prosopagnosics” (Jones & Tranel, 2001). And more recently still, attention has been drawn to “super-recognisers”, who lie at the top end of performance and are exceptionally skilled at face memory tasks (Russell, Duchaine, & Nakayama, 2009). But as most people’s face recognition ability lies in the range between that of a prosopagnosic and a super-recogniser, until quite recently it is this “average” performance that most theories of face recognition have tried to explain. Previously, little attention has been paid to individual differences, apart from the nature of the deficits that could account for neuropsychological impairments in acquired prosopagnosia (see Bruce & Young, 2012, for a general introduction).

Before looking at the applied implications of individual variation in face identification ability in two specific operational contexts (passport control and identification in criminal situations), we first consider what we know about performance in the laboratory. Specifically, we outline how an individual-differences approach can be used to illuminate the neurological underpinnings of face identification, and the linkages, or otherwise, between face identification and other aspects of cognitive and social processing. Here we pose a number of questions to explore the possible relationship between face identification ability and structural/neural differences in the brain, general cognitive factors, social factors and holistic processing ability. Rather than simply showing a relationship between factors, we aim to show how studies utilising an individual-differences approach can add to our theoretical knowledge of the mechanisms underpinning face identification and can offer perspectives on face processing that may be missed using a group-mean approach.

### Do individual differences reflect structural or processing differences in the brain?

One possibility is that individual differences in face identification reflect structural or processing differences in the brain, and thereby enhance our understanding of the neurological underpinnings of face processing. Over the past two decades a network of “core” brain areas have been linked to face perception, in particular the fusiform face area (FFA), occipital face area (OFA) and superior temporal sulcus (STS). The FFA is thought to be involved in the processing of non-changeable aspects of faces, such as gender and face familiarity (Kanwisher, McDermott, & Chun, 1997). The OFA is considered to be responsible for the early processing of facial stimuli and facial parts (Pitcher, Walsh, & Duchaine, 2011). Finally, the STS appears to be involved in the processing of “changeable” aspects of faces, such as eye gaze and emotional expression (Hoffman & Haxby, 2000). The identification of these different face-specific areas in the brain led to the development of the neural model of face processing by Haxby, Hoffman, and Gobbini (2000); this was later modified by Gobbini & Haxby (2007) in the distributed model of face processing.

There is limited work exploring the relationship between individual differences in face identification and associated brain structure and neural processing differences. In early work, Schretlen, Pearlson, Anthony, and Yates (2001) suggested that the ventricle-to-brain ratio accounted for 25% of individual variance in a face discrimination task. Rotshtein, Geng, Driver, and Dolan (2007) showed that individual variation in sensitivity to configural changes in faces was correlated with face recognition performance, with self-rated face recognition ability, and also with measures of brain activation of face-related areas, such as the fusiform and occipital gyrus. More recent work has found links between regional grey matter volume in the brain and face recognition deficits in those with schizophrenia (Onitsuka et al., 2005) and developmental prosopagnosia (Garrido et al., 2009; also see work on functional magnetic resonance imaging (fMRI) differences by Gomez et al., 2015; Song et al., 2015; Tavor et al., 2014). In healthy participants, Li et al. (2016) found that grey matter volume of the right ventral anterior temporal lobe is positively correlated with face recognition ability, as measured using the Cambridge Face Memory task (Duchaine & Nakayama, 2006). Interestingly, the grey matter volume of other brain areas linked to face recognition - the FFA (Kanwisher et al., 1997) and the OFA (Pitcher et al., 2011) - was not significantly correlated with face identification performance.

Instead of exploring the amount or nature of brain tissue, other research has focused on investigating differences in connectivity between brain regions in the face processing network. For example, Rosenthal et al. (2017) used this approach to investigate differences between individuals with inherited developmental prosopagnosia (termed “congenital prosopagnosia”) and healthy individuals. They found that in healthy participants the anterior temporal cortex was highly connected to other face processing areas, whereas this connectivity was considerably reduced in participants with congenital prosopagnosia (also see Avidan & Behrmann, 2014; Lohse et al., 2016). It would be useful for future work to explore both brain connectivity and “hyperconnectivity” (see Rosenthal et al., 2017) in relation to individual face processing ability.

A number of studies have used event-related potentials (ERPs, see Luck, 2005) to explore the neural mechanisms underpinning individual differences in face perception and memory. Here, performance has been related to the neural amplitudes and latencies of ERPs (Luck, 2005). Several neural components have been identified that are associated with face processing. First, the N170 is a negative peak located occipito-temporally that occurs around 150–190 ms after presentation of a face. The N170 amplitude is typically larger for faces than for other objects (Bentin, Allison, Puce, Perez, & McCarthy, 1996) and is thought to be related to the creation of face representations (Eimer, 2011) rather than face familiarity (Herzmann, Schweinberger, Sommer, & Jentzsch, 2004). Second, the N250r is found temporo-parietally at 260–330 ms post face presentation. It is thought to originate in the FFA (Schweinberger, Pickering, Jentzsch, Burton, & Kaufmann, 2002) and reflect familiarity-processing of faces (Schweinberger & Burton, 2011). Finally, the N400, a neural response located parietally between 300 and 500 ms has been linked to the activation of semantic person-related knowledge stored in long-term memory (Schweinberger & Burton, 2011).

Interestingly, Kaltwasser, Hildebrandt, Recio, Wilhelm, and Sommer (2014) found that face identification performance (accuracy and speed) is related to the speed of structural encoding (latency of the N170 response) and with access to familiarity and semantic information about faces (N250r and N400 latencies and amplitudes). Very recent work has compared neural indicators for face and object processing recognition in order to explore the specificity of the observed links between variations in ERPs and performance, and reported that the N170 response has significant face-specific variance (Rostami, Sommer, Zhou, Wilhelm, & Hildebrandt, 2017; also see work by Towler, Fisher, & Eimer, 2017 on ERP differences between individuals with developmental prosopagnosia and controls).

Thus, in addition to evidence showing that damage to some brain regions is associated with prosopagnosia (e.g., see Barton, 2008), within the normal range of face processing performance there is also some evidence to support the relationship between brain structural differences, connectivity and neural processing differences and individual differences in face perception and memory. However, much more work is needed to consider the extent and impact of such a relationship.

### Do individual differences reflect general cognitive processing?

A second (not mutually exclusive) possibility is that individual differences in face identification reflect the general cognitive processing of an individual rather than being specific to face identification per se (for an explanation of “cognitive specificity” see Wilmer, 2017). Interestingly, individual differences in face processing do not seem to be mere reflections of IQ. Indeed, recent work has suggested that IQ only explains 3 or 4% of variation in face identification performance (see Gignac, Shankaralingam, Walker, & Kilpatrick, 2016; Shakeshaft & Plomin, 2015; Van Gulick, McGugin, & Gauthier, 2016; Wilmer et al., 2010; Wilmer, Germine, & Nakayama, 2014). In addition, there are known to be some genetic components to face identification ability (see Shakeshaft & Plomin, 2015).

Further work has looked at the relationship between face identification ability and other aspects of cognitive performance. Here, there does seem to be some relationship between face processing and general visual processing ability. For example, performance in an unfamiliar face-matching task has been related to visual short-term memory, perceptual speed and object matching ability (Megreya & Burton, 2006), as well as perceptual style (Hoffman & Kagan, 1977; Witkin & Goodenough, 1977), object matching (Burton et al., 2010) and space perception (Burgess, Alderman, Evans, Emslie, & Wilson, 1998). Work by Dennett et al. (2012) also identified moderate but significant correlation between performance on the Cambridge Face Memory Test and the Cambridge Car Memory Test.

These findings are interesting but different relationships are found depending on the face task included and the cognitive performance measures used. A more global approach has been adopted in recent individual-differences studies conducted by Wilhelm and colleagues (for example, Hildebrandt, Wilhelm, Schmiedek, Herzmann, & Sommer, 2011; Wilhelm et al., 2010, 2012). Wilhelm et al. (2010) investigated performance across a number of face perception, face memory and cognitive processing tasks. Specifically, in Experiment 1, 151 participants completed a set of tasks designed to test face “cognition”; five on face perception accuracy (simultaneous and sequential matching; facial resemblance), three on face memory accuracy (acquisition and delay of learned faces; eyewitness testimony) and six on speed of face cognition (recognition speed; speed in delayed non-matching to sample task; matching speed). Structural equation modelling was used to test a theoretically driven measurement model that made a distinction between face perception and face memory, and between accuracy and speed, and compared this account against other competing models. Results supported a distinction between accuracy and speed of face cognition. Furthermore, for face accuracy there was a distinction between face perception and face memory (Wilhelm et al., 2010; but see recent work by Verhallen et al., 2017, who propose a global face perception factor ‘*f*’ to encompass performance on the Cambridge Face Memory Test and the Glasgow Face Matching Test). In Experiment 2, Wilhelm et al. (2010) investigated the relationship between these identified aspects of face cognition and other aspects of cognition (*N* = 209). Participants were additionally asked to take part in tasks measuring object cognition, immediate and delayed memory, general cognitive ability, and mental speed. Results replicated the findings from Experiment 1 and found that individual differences in face cognition could not be reduced to individual differences in object cognition, memory, general cognitive ability or mental speed.

It seems that the evidence is, as yet, still inconclusive, with some researchers proposing a link between specific aspects of face identification and cognitive ability (see for example, Megreya & Burton, 2006). However, here the reliance is on a single task, which makes it difficult to disentangle the importance of task specificities from the conclusions drawn. In contrast, research using structural equation modelling has utilised larger numbers of participants and instead focused on commonalities across groups of tasks. Here, the evidence suggests that individual differences in face identification cannot be explained by more general cognitive processing differences.

### Do individual differences reflect personality and social interaction?

Differences in aspects of personality and social interaction have also been linked with face identification ability. Here we do not consider disorders of development, like Autistic Spectrum Disorder (ASD) or Williams Syndrome (WS), where links between face perception and wider aspects of social cognition have been made (see, for example, Weigelt, Koldewyn, & Kanwisher, 2012; Karmiloff-Smith et al., 2004, respectively). Rather, we consider more specific aspects of personality. For example, Bate, Parris, Haslam, and Kay (2010) investigated the association between empathy and face processing. Empathy is a “concept consisting of our ability not only to share emotions but also to exert cognitive control and perspective taking in our [social] interactions with others” (Banissy, Kanai, Walsh, & Rees, 2012, p. 2034). Bate et al. (2010) found that those high in empathy performed significantly better at a face recognition memory task than those with low empathy skills.

Anxiety has also been associated with individual differences in face processing ability. Early work by Mueller, Bailis, and Golstein (1979) compared the face recognition ability, which was assessed with a recognition memory task, of participants reporting high and low levels of general anxiety on the Test Anxiety Scale. They found significantly better face recognition performance in those low in general anxiety (see Deffenbacher, Bornstein, Penrod, & McGorty, 2004, for work on anxiety and eyewitness recognition; also see Martin, Hancock, & Frowd, 2017, on stress and face composite creation). In similar classic work, Nowicki, Winograd, and Millard (1979) found there was positive correlation between trait anxiety and overall anxiety (sum of state and trait anxiety) with face recognition performance in female, but not male individuals. In more recent work, Davis et al. (2011) identified a small but significant relationship, whereby poorer face recognition on the Cambridge Face Memory Test was associated not with general anxiety but with higher social anxiety.

Finally, in terms of anxiety, Megreya and Bindemann (2013) tested correlation between performance on a face line-up matching task (see Megreya & Burton, 2006) and Cattell and Cattell’s Sixteen Personality Factor Questionnaire (Cattell & Cattell, 1995), which measures the five global personality factors of extraversion, anxiety, tough-mindedness, independence and self-control. A second experiment then combined the same face task with the NEO Big-Five Personality Inventory (Costa & McCrae, 1992), which measures neuroticism, extraversion, openness to experience, agreeableness, and conscientiousness. In this personality inventory, “anxiety” is one of the six facets of neuroticism. Experiment 1 identified negative correlation between correct identifications and anxiety in female participants, but not in male observers. Here, anxious female observers were less likely to correctly identify the face targets from the line-ups. Experiment 2 replicated the correlation between correct face identifications and anxiety in female participants. These results suggest that associations between personality and face perception are limited and are confined to anxiety and facets of neuroticism (also see Hills, Eaton, & Pake, 2016; Megreya & Bindemann, 2013, who found no link between face recognition and extraversion but see other work detailed below). Socially anxious individuals may avoid social interactions (see Bögels et al., 2010), leading to less exposure to faces, accounting for the specific reduction in face recognition ability.

A number of studies have also explored the relationship between extraversion and individual face recognition ability. Li et al. (2010) found that extraverts with enhanced social skills performed better than introverts in a face recognition memory task (but see early work by Thompson & Mueller, 1984). Extraverts are known to be superior at decoding social information and to be more involved in social activities than introverts. Indeed, Fishman, Ng, and Bellugi (2011) using ERP methodology, found that social stimuli carry enhanced motivational significance for extraverts and that this is related to individual differences in neural responses to social stimuli. Specifically, Li et al. (2010) identified positive correlation between face recognition ability (difference in accuracy in recognition of faces and flowers) and extraversion, with gregariousness being particularly important. This relationship was independent of IQ, as measured by Raven’s matrices. Follow-up work by Lander and Poyarekar (2015) identified positive correlation between extraversion and performance in a famous face recognition task, but no such relationship between extraversion and unfamiliar face matching (Glasgow Face Matching Test; Burton et al., 2010).

We can see that a relationship between various aspects of social interaction, personality and face identification has been found. Further work is needed to investigate the theoretical underpinnings of these findings. For example, if the link between extraversion and face recognition is causal then an individual’s inability to learn and recognise faces may lead them to become more introverted, to avoid potentially embarrassing social situations - or introverted individuals may meet fewer people and thus never develop good face recognition skills. Similarly, good face recognition skills may lead to an individual developing a more extraverted personality. A further possibility is that causality is bidirectional or that both extraversion and face recognition are related to another as yet unidentified factor.

Considering the direct importance of faces for interactions between people (see, e.g., Hari & Kujala, 2009), “social interaction” seems like a possible overarching factor that could link individual differences in face identification with those in empathy, social anxiety and extraversion. Indeed, whilst much of the previous work investigates a single personality trait, more consideration is needed to determine how these findings fit together into an integrative theory (Megreya & Bindemann, 2013) incorporating the wider factor of social interaction. In addition, we need to consider how much practical impact these issues have on face identification in applied situations.

### Do individual differences reflect variations in holistic processing?

The idea of holistic processing of faces has been prevalent in the face recognition literature for many years (see early work by Young, Hellawell, & Hay, 1987), with this typically meaning that faces are represented in memory as a whole with little or no explicit representation of parts (see Farah, Wilson, Drain, & Tanaka, 1998; Maurer, Le Grand, & Mondloch, 2002; Piepers & Robbins, 2013). Holistic processing has predominantly been measured using the composite face task (Rossion, 2013; Young et al., 1987) and the part-whole task (Tanaka & Farah, 1993). There has been some debate in the literature about whether these tasks adequately measure holistic processing (McKone et al., 2013; also see Rossion & Fetter, 2015) and whether they measure the same thing (see DeGutis, Wilmer, Mercado, & Cohan, 2013, for correlation of *r* = 0.44 between the composite and part-whole tasks) or reflect different aspects of face processing (see Wang, Li, Fang, Tian, & Liu, 2012, for correlation of *r* = 0.03). In the composite task of Young et al. (1987), the top (or bottom) half of a famous face was named faster when misaligned with the bottom (or top) half of a different famous face, compared to when the top and bottom halves were lined up to form a “new” face. The difference between the aligned and mis-aligned conditions was only found for upright faces, not inverted ones. A similar pattern of results is found when unfamiliar faces are used in the “composite” task and the participant is asked to focus on the top half of the faces and decide if the two target regions (e.g., top halves) are identical or not (Hole, 1994). In the part-whole task, Tanaka and Farah (1993) found that participants were better able to decide “Which is Larry’s nose?” when the nose feature was presented within the context of the whole face rather than when presented alone.

Importantly, researchers have suggested that holistic processing ability underpins our face identification ability (Maurer et al., 2002). Indeed, it has been found that some prosopagnosic participants are impaired at holistic processing as measured using the composite task (see, for example, Avidan, Tanzer, & Behrmann, 2011; Palermo et al., 2011 but see Biotti et al., 2017; Susilo et al., 2010; Ulrich et al., 2017). However, the evidence for holistic processing being a source of individual differences in face identification performance is somewhat mixed. On the one hand, some research has failed to find any relationship between holistic processing and face identification ability. For example, Verhallen et al. (2017) tested face processing using the Mooney Face test, Glasgow Face Matching Test (GFMT; Burton et al., 2010), Cambridge Face Memory Test (CFMT; Duchaine & Nakayama, 2006) and a composite face test. No correlation was found between holistic processing (as measured using the composite test) and any aspect of face processing (also see work by Konar, Bennett, & Sekuler, 2010). In recent work, Li, Huang, Song, and Liu (2017) demonstrated dissociation in the neural basis between the composite face task (Young et al., 1987) and the part-whole task (Tanaka & Farah, 1993). They found that the composite face task was correlated with face selectivity in the right FFA whereas the part-whole task (termed “whole-part” by Li et al., 2017) was correlated with face selectivity in the left FFA. Li et al. (2017) thus support the idea that the composite task and the part-whole task reflect different aspects of holistic face processing.

In contrast, other work has identified significant positive correlation between holistic processing and face recognition (composite task and Cambridge Face Memory Task, Richler, Cheung, & Gauthier, 2011; part-whole task and Cambridge Face Memory Task, Degutis et al., 2013). Degutis et al. (2013) proposed that research in this area has been hampered by the manner in which holistic processing has been calculated (by subtracting the control condition from the condition of interest, leading to confounding variables) and proposed a new method (by regressing the control condition from the condition of interest, leading to independent variables). The results showed that there was correlation between performance on a part-whole and composite task when using the regression-based holistic processing measure, supporting the idea of a unitary construct of holistic processing. Furthermore, holistic processing was clearly linked to individual face recognition ability (using the Cambridge Face Memory Task; Duchaine & Nakayama, 2006). More work is needed to explore both the link between different holistic processing tasks and the relationship between holistic processing and individual face recognition performance, both in neural and behavioural terms.

We can see then that during the last 10 years or so, interest in individual differences in face identification has been steadily rising. Research in the laboratory has linked individual differences to structural and processing differences in the brain, with better performance linked to increased grey matter volume (Li et al., 2016), neural connectivity between face-areas (Rosenthal et al., 2017) and the N170 neural response (Rostami et al., 2017). Further work has focused on the links between individual differences in face identification ability and other cognitive processing abilities, with larger scale studies finding little relationship between face perception, recognition and general cognitive processing, thus supporting the idea of face-specifity (e.g., Wilhelm et al., 2010; Wilmer et al., 2010). Clear relationships between individual face recognition ability and empathy (Bate et al., 2010), social anxiety (Davis et al., 2011) and extraversion (Li et al., 2010) have been identified, which are all factors that may be related to the wider construct of social interaction. Finally, individual differences in face recognition have been related to holistic processing with mixed findings (Degutis et al., 2013; Verhallen et al., 2017). It is possible that participants possess all or just some of the characteristics that are associated with better or worse face identification skills.

Whilst it is important to consider individual differences theoretically in the laboratory, we also need to relate our findings operationally to face identification in real applied situations. Here, we focus on individual differences that may impact upon professional roles that involve face identification, and we have omitted non-professional roles where individual differences may also be critical, particularly where eye-witnesses may be asked to identify perpetrators from photographs and line-ups. This is an important area that is also beginning to attract attention (e.g., see Bindemann, Brown, Koyas, & Russ, 2012; Morgan et al., 2007).

In the real world, there are a number of job roles where face identification is a key skill, such as passport control officers who match a person to a passport photo, police officers identifying suspects, and other security and customer-facing roles. However, currently an individual’s face identification ability does not formally constitute part of the recruitment process to these roles. We first consider the case of passport control officers (or other gate-keepers who use photo-ID to verify identity). Here the task is to match the person appearing in front of them to the individual shown on a passport ID picture. We review research that has been conducted on this face-matching task and consider the role of individual variation in ability. A challenge occurs when the individual making the facial identification decisions is poor at this task. We outline research that has been conducted on face training and consider whether this would be beneficial in improving matching performance.

## Case 1: passport control - face identity matching and training

To permit a person entry to other countries, some form of identity verification is typically required. Although there is increasing interest in different biometric markers to support this entry-to-country process (including fingerprint scanning, retina scanning, iris recognition, finger geometry, voice recognition, DNA matching and gait analysis), the most prevalent means of identification is still from the verification of facial photo-ID. Indeed, although automated face recognition systems are increasingly in use for person identification at airports and borders, these systems operate under the supervision of human operators, who can intervene and override automated decisions (see FRONTEX, 2015). Whether using automated face recognition systems or direct verification of photo-ID by a human observer, this identification task typically relies on trained passport control specialists to perform this task accurately and quickly (White, Kemp, Jenkins, Matheson, & Burton, 2014).

In the past, people seeking to enter a country illegally at passport control may have utilised fraudulent identity documents, into which their photo has been inserted. However, modern passports are difficult to manipulate in this way. An alternative method to gain entry to a country, which avoids the kind of tampering with identity documents that could be detected at passport control, is to use the passport of another person that is of similar facial appearance. The role of the passport officer then is one of face matching - matching the live person in front of them, or a still image provided by an automated recognition system, to the individual shown on their passport documentation - with the aim of detecting identity fraud, when the face depicted in a photo-identity document does not match its bearer.

This task is referred to as unfamiliar face matching and has received considerable attention in recent years (for a review, see Fysh & Bindemann, 2017). Research has shown that face matching is error-prone under highly optimized conditions, such as when observers are asked to compare photographs of the same person that were only taken minutes apart, and are highly similar in terms of lighting, facial expression and pose (see Burton et al., 2010). Accuracy declines further as this task more closely approximates the demands of operational settings, such as passport control. For example, passports are typically valid through a 10-year period but a person’s facial appearance varies over time (e.g., through changes in hairstyle, weight, adiposity). The resemblance of a person to their passport photograph therefore changes also. This is reflected in identification accuracy, which declines by around 20% when matching of same-day photographs is compared with photographs taken even just several months apart (Megreya, Sanford, & Burton, 2013). Embedding face photographs in identity documents also produces an identification bias, which specifically affects detection of the crucial identity mismatches (McCaffery & Burton, 2016). This bias is also found when observers are asked to match many faces over long periods (Alenezi & Bindemann, 2013; Alenezi, Bindemann, Fysh, & Johnston, 2015), which is the norm at passport control.

These studies highlight the challenging nature of this identification task but are typically based on group performance. This is remarkable given that individual differences appear to provide an enormous source of error in face matching. Under best-case conditions, for example, individual performance ranges from seemingly at random (i.e., at around a 50% chance level) to perfectly accurate (100%; Burton et al., 2010), even in experiments comprising relatively small samples (e.g., Estudillo & Bindemann, 2014). These findings have been obtained with student participants, who were untrained in facial identification. However, a few studies have directly examined the performance of passport control officers. For example, White, Kemp, Jenkins, Matheson, and Burton (2014) investigated the identification accuracy of passport officers with a mean of 8 years working in the job, across a range of face-matching tasks including person-to-photo and photo-to-photo matching. As a group, the passport officers had high error rates when making matching decisions and throughout performed comparably to student participants who had received no training and had no professional experience in person identification. This indicates that the training of the passport officers was not effective in improving face-matching performance.

An even more striking picture emerged when individual differences in the matching accuracy of these passport officers were examined. In the Glasgow Face Matching Test, for example, passport officers exhibited an enormous range in individual accuracy, from below 60% to 95%. The lowest accuracy was recorded by an officer with over 20 years’ experience, whereas the highest score was obtained by one of the least experienced individuals, demonstrating also that experience is a poor indicator of face matching performance (for similar findings, see Wirth & Carbon, 2017). Moreover, whereas such passport officers can be outperformed at a group level by expert facial examiners, who are specialist in identity comparisons in legal settings, even these specialists demonstrate a very broad range in individual face-matching performance (see White, Dunn, Schmid, & Kemp, 2015).

What then are the implications of these findings? First and foremost, the available evidence demonstrates clearly that professionals such as passport officers are likely to vary substantially in their face matching ability. This raises questions about the effectiveness of personnel selection procedures and also must carry a security cost for applied settings. Moreover, the research evidence suggests common-sense indices, such as experience (White, Kemp, Jenkins, Matheson, & Burton, 2014) or specialist training and expert status (White, Dunn, et al., 2015; White, Phillips, Hahn, Hill, & O'Toole, 2015) do not appear to act as effective predictors of individual ability. In addition, people seem to have limited insight into their own face perception abilities (see Bindemann, Attard, & Johnston, 2014; Bobak, Mileva, & Hancock, in press; Palermo et al., 2017; but see Gray, Bird, & Cook, 2017; Livingston & Shah, in press; Shah, Gaule, Sowden, Bird, & Cook, 2015). This implies that experts’ self-reports also cannot be taken as evidence that they possess enhanced ability to identify faces (see also Austen, Bindemann, Griffiths, & Roberts, 2018).

However, whilst observers may lack insight into their face identification ability generally, it is possible that the confidence in their identification decisions, on a trial-by-trial basis, may be a useful indicator of their identification accuracy. Eyewitness identification research has extensively investigated the relationship between confidence and accuracy, with witnesses often knowing that they are at risk of making an error. This is then communicated by expressing low confidence in their identification decision (see Wixted & Wells, 2017). Similarly, Stephens, Semmler, and Sauer (2017) suggest that individuals’ confidence in their face matching decisions may be an indicator of their accuracy, whereby high-confidence decisions tend to be accurate and low-confidence decisions not. Whether this relationship extends to professionals, such as passport officers, remains to be seen.

In the meantime, individual differences in psychological experiments indicate that identification accuracy in security settings, such as at passport control, could be maximized by selecting individuals by their face-matching ability. However, a number of key questions remain here before such a recruitment strategy could be adopted in the field. For example, it remains unknown at present whether performance on a laboratory-based face matching task correlates strongly with passport control verification in the field. Correlation between matching a live person to photo and photo-to-photo matching may speak to this issue, but have so far yielded insufficient data to draw firm conclusions (White, Kemp, Jenkins, Matheson, & Burton, 2014).

Another key question concerns whether matching performance is stable within an individual. Whereas some individuals display consistently high accuracy in face matching across different days, many also exhibit notable variation. This is such that some observers can perform with high accuracy on one day but not on another, or on one block of trials but not on the next (Bindemann, Avetisyan, & Rakow, 2012). This problem is compounded as observers also seem to exhibit variation in their responses to the *same* stimuli within a single testing session. For example, when face stimuli are repeated across blocks in a prolonged matching experiment, the detection of identity mismatches declines continuously to near-chance level (see Alenezi & Bindemann, 2013). This behavior reflects a change in responses to the same face stimuli over time and resists simple explanation (see Alenezi et al., 2015). Importantly, however, it also suggests that selection of individuals for professional roles by their face-matching ability cannot be achieved with a “quick” test, but must likely involve thorough testing over a prolonged period. Finally, further evidence is also required that individuals’ face-matching performance is stable across faces of different races. Psychological research has demonstrated consistently that people are worse at identifying faces of an ethnic background different to an observer’s own (see Meissner & Brigham, 2001), but passport officers are required to identify people accurately irrespective of ethnicity. Some evidence is emerging to demonstrate that individual differences in the ability to identify faces transcend race, such as correlations between same-race and other-race identification in one-to-many (Megreya, White, & Burton, 2011) and one-to-one matching tasks (Kokje, Bindemann, & Megreya, 2018). In addition, correlation between performance in different-race versions of the Cambridge Face Memory Test has also been identified (Wan et al., 2017). However, these studies are based on very few races, such as comparison of Arab and Caucasian faces. Overall, sufficient data on individuals’ ability to match faces from many different races still does not exist.

Finally, it remains unresolved which task should be used as part of the recruitment process for security professions that rely on face matching. For psychological research, for example, some face tests provide highly optimized conditions to capture the best possible matching accuracy (Burton et al., 2010), whereas other tests are more challenging by capturing the variation that people may exhibit over time (Fysh & Bindemann, 2018). There is moderate correlation between performance in these tests (*r* = 0.45). Moreover, even when performance between different matching tests correlates strongly, key individuals can exhibit important variation across these. For example, Bobak, Dowsett, and Bate (2016) showed that among a group of seven people with extraordinary face recognition ability, three outperformed a control group at an individual level on one face matching test, and five outperformed the control group on a different face matching test, but only two of these seven individuals displayed such exceptional performance on both tests. Taken together, these data indicate that application of face matching tests for personnel selection will produce different results depending on which test is used. This suggests that such personnel selection might ultimately require complex and time-consuming solutions that are based on combinations of various tests, but the precise nature of the appropriate tests and their respective combinations remains unclear.

An alternative possibility is that we try and identify officers who are poor at face matching in the laboratory and help them improve their matching performance through training. Here, the hope would be that training in face-to-face matching will translate to different contexts and improve matching decisions in the field. So far, work on face-matching training has had mixed results. Early research by O'Toole et al. (2007) showed that practice without feedback was not useful for improving face-matching performance. Similarly, training participants to make matching decisions based on face shape was not beneficial (Towler, White, & Kemp, 2014). However, training with trial-by-trial feedback has been shown to be helpful for improving accuracy in both sequential (Hussain, Sekuler, & Bennett, 2009) and simultaneous matching tasks (White, Kemp, Jenkins, & Burton, 2014; also see work by Chen & Liu, 2009; Liu, Bhuiyan, Ward, & Sui, 2009), and can also help to maintain accuracy when it would otherwise decline during repetitive testing (Alenezi & Bindemann, 2013). Forcing participants to compare facial features prior to making a match decision has also been shown to improve accuracy (Towler, White, & Kemp, 2017), as have instructions to attend to specific facial features (Megreya & Bindemann, 2018), highlighting further potential training strategies. It is less clear to what extent such training effects translate to the matching of new faces, or faces from a different stimulus set, rather than reflecting the learning of previously viewed faces or their facial characteristics (see, for example, Bruck, Cavanagh, & Ceci, 1991), and whether any performance gains will persist long term. Moreover, some of the most promising effects, such as improvements with feedback or feature instruction, have been obtained with experiments comprising very few trials (see Megreya & Bindemann, 2018; White, Kemp, Jenkins, & Burton, 2014). Thus, it seems likely that such manipulations enhance performance by helping observers refine their *criteria* for distinguishing identity matches from mismatches (i.e., by drawing attention to specific features), rather than by improving their cognitive *ability* to process faces better (i.e., by helping to extract more information from a looked-at feature). So far, there have been few attempts with more extensive training protocols that span several days and might improve ability. These have shown only limited improvement from before to after training (of 5%) and no generalization to untrained identities (Matthews & Mondloch, in press). Clearly, then, much further research is needed to develop successful training techniques that can produce lasting improvements in individuals’ matching accuracy and that can be generalised to complex real-world settings. This is an important avenue for further research.

## Case 2: police officers - identification and super-recognition

The aforementioned research on passport officers demonstrates that security personnel can be poor at facial identification, even when this is central to their professional duties. We note that these findings may also extend to police officers. At German airports, for example, passport control is, in fact, populated by Federal Police officers. These police officers are also prone to errors in face matching and also display substantial individual differences (Wirth & Carbon, 2017). However, an opportunity occurs when we strategically place police officers who are already skilled at face recognition into operational roles that make use of this ability. Here, recent work has looked at “super-recognisers” (see review by Noyes, Phillips, & O'Toole, 2017). This term was first used by Russell et al. (2009), who described four participants who self-presented with extraordinary face recognition ability. All of these super-recognisers performed better than any of 25 control participants on tests of familiar face recognition (Before They Were Famous test) and unfamiliar face learning (Cambridge Face Memory Test; Duchaine & Nakayama, 2006; over two standard deviations above the mean is sometimes cited as being “diagnostic” of super-recognition).

Such super-recognisers are now identified and deployed within the police. In the London Metropolitan police (UK), for example, officers who are believed to have an exceptional ability to recognise people have been used to identify large numbers of criminal perpetrators from challenging surveillance footage, such as that of the 2011 London riots. This has attracted considerable attention in the media (e.g., see The New Yorker, 2016). How do these officers perform in scientific tests of their face identification ability? When tested using controlled face identification tasks, an investigation of four super-recognisers at the London Metropolitan police showed that they consistently outperformed police trainees and student observers in an optimized test and a challenging test of unfamiliar-face matching, and in the identification of familiar (celebrity) faces from heavily pixelated footage (Robertson, Noyes, Dowsett, Jenkins, & Burton, 2016). In another study, comprising a much larger sample of 35 super-recognisers from the London Metropolitan police, these professionals also outperformed controls across recognition tests for famous faces, memory tests for unfamiliar faces and in unfamiliar face matching (Davis, Lander, Evans, & Jansari, 2016). Moreover, these police officers achieved similar performance to ten super-recognisers, who were selected from the general public for their exceptional face processing ability. However, whilst these data suggest that these specialist police officers are exceptionally good at face processing at a group level, a different picture emerges when the individual data are considered. In the two memory tests, for example, less than a third of police super-recognisers outperformed the mean performance of an untrained control group, whilst the matching test lacked sensitivity to distinguish high-performing individuals from controls. Overall, the psychological data therefore suggest that individuals with exceptionally high face recognition ability can be selected for, or already inhabit, some key roles in the police for which these skills are important. At the same time, not all police super-recognisers appear to be super at face recognition.

A key issue that emerges from this work is *how* the successful super-recognisers might differ from other observers. One possibility is that these individuals are perhaps more motivated to perform well. This seems unlikely considering that there are broad individual differences in performance among individuals from the same professional groups, and that performance of the same individuals can differ across tests (see, e.g., Cohan, Nakayama, & Duchaine, 2016; Davis et al., 2016; White, Kemp, Jenkins, Matheson, & Burton, 2014). Similarly, it appears unlikely that a performance advantage arises from the amount of time that observers dedicate to the task at hand, as longer response times (at a group level) in unspeeded matching tasks do not seem to yield a performance advantage (see White, Kemp, Jenkins, Matheson, & Burton, 2014; but see also White, Phillips, et al., 2015). Further work has investigated whether super-recognisers look at faces differently or make different use of facial features compared with control participants. This research, which has focused on the free viewing of images of people in social scenes and a face learning task, suggests that super-recognisers spend more time fixating the centre of faces (i.e, the noses; also see Bennetts, Mole, & Bate, 2017), which might allow these observers to spread spatial attention more effectively across faces (Bobak, Parris, Gregory, Bennetts, & Bate, 2017). However, this was the only facial region that was afforded such a privileged status, and only by a proportion of the super-recognisers under investigation.

Whilst additional eye-tracking work is needed to explore individual differences in the strategies adopted during face viewing, heterogeneity in performance by individual super-recognisers has now been observed in several studies (e.g., Bobak, Bennetts, Parris, Jansari & Bate, 2016; Bobak et al., 2017; Davis et al., 2016). This indicates that super-recognition might not represent a unitary ability advantage in face processing, but that specific facets of super-recognition might present in individual observers depending on which task is at hand. Bobak, Hancock, and Bate (2016) found, for example, that four out of seven super-recognisers outperformed controls in a facial identity matching task, but only one of these also showed an advantage in a face memory task. This suggests that some aspects of super-recognition may be related to the heightened perceptual processing of faces, whereas others might reflect identity-specific memory for faces. It may be that different “super” abilities can be best utilized in different roles: with “super-face-memorisers” helping to identify known individuals (as outlined in the police or in specific security situations like identifying banned fans from football matches) and “super-face-matchers” more useful in the verification of identity (for example, at passport control, as previously discussed).

Related to this context, a further question concerns whether such super-recognition abilities are related specifically to faces. Some tentative evidence for such specificity comes from two world memory competitors, who were highly skilled at memory tasks but did not differ from control participants in their face processing and recognition abilities (Ramon et al., 2016). Other research suggests that many visual and non-visual abilities are strong in super-recognisers (Duchaine, 2017). Further studies are needed to understand the specificity of extraordinary face skills.

## Conclusions

Whereas much research on individual differences in face matching and face recognition is motivated directly by practical problems facing security staff, such as passport officers and police, this research has direct theoretical implications. Cognitive models of face recognition (e.g., Bruce & Young, 1986; Burton, Bruce, & Johnston, 1990; Burton, Jenkins, Hancock, & White, 2005) or face detection (Lewis & Edmonds, 2005) typically do not take account of individual differences except for specific neuropsychological cases that reveal something about the general organisation of these systems, such as dissociations in the analysis of facial expressions from the process of face recognition. Similarly, these models only capture individual differences to a very limited extent. For example, some theories explain neuropsychological cases that are marked by impairments in aspects of face processing via residual activation in the typical face processing system (see Schweinberger & Burton, 2003). None of these theories, however, capture the individual differences that are observed among neurologically normal observers, and which are the focus of this special issue. This is remarkable as individual differences appear to be one of the largest - if not the largest - documented potential source of error in face matching and face recognition. Moreover, the *individual* ultimately provides the ‘hardware’ within which any cognitive face processing system resides. In this respect, studies of individual differences in face matching and face recognition with a predominantly applied focus provide a strong reminder that it would be remiss not to incorporate individual differences into theoretical models. However, as the examples reviewed here also demonstrate extensively, it is relatively straightforward to chart individual differences across various populations and face-processing tasks, but our theoretical understanding of these differences has not advanced at the same rate. Consequently, we still cannot provide convincing accounts of why some people are worse than others in seemingly simple tasks, such as pairwise face matching, nor do we possess robust theoretical frameworks to inform training programmes for improving the face skills of poorly performing individuals.

## Footnotes

1 Note: In this paper we draw a distinction between face recognition and face matching. “Recognition” is used to refer to tasks that require memory of a newly learned face, which is then recognised, after an interval, at a subsequent test phase. “Matching” refers to tasks requiring identity comparisons of simultaneously presented unfamiliar faces, but do not require memory of these faces for subsequent recognition. We use “identification” as an umbrella term to capture both of these processes - recognition and matching.
